# ﻿Two new species of *Meliola* (Meliolales, Sordariomycetes) from Guizhou Province, China

**DOI:** 10.3897/mycokeys.122.161471

**Published:** 2025-09-15

**Authors:** Yuzhe Feng, Yuwei Liu, Sinang Hongsanan, Entaj Tarafder, Xiaolu Deng, Tian Yang, Jipeng Sun, Ting-Chi Wen, Xiang-Yu Zeng, Fengquan Liu

**Affiliations:** 1 Department of Plant Pathology, College of Agriculture, Guizhou University, Guiyang, 550025 China Guizhou University Guiyang China; 2 Guizhou Key Laboratory of Agricultural Microbiology, Guiyang, 550025 China Guizhou Key Laboratory of Agricultural Microbiology Guiyang China; 3 Institute of Edible Mushrooms, Guizhou University, Guiyang, 550025 China Shenzhen University Shenzhen China; 4 Shenzhen Key Laboratory of Microbial Genetic Engineering, College of Life Science and Oceanography, Shenzhen University, Shenzhen 518060, China Guizhou University Guiyang China; 5 State Key Laboratory of Green Pesticide, Key Laboratory of Green Pesticide and Agricultural Bioengineering, Ministry of Education, Guizhou University, Guiyang 550025, China Guizhou Key Laboratory of Agricultural Microbiology Guiyang China; 6 Engineering Research Center of Southwest Bio-Pharmaceutical Resources, Ministry of Education, Guizhou University, Guiyang 550025, China Shenzhen University Shenzhen China

**Keywords:** Black mildews, fungal diversity, new species, phylogenetic analysis, taxonomy

## Abstract

*Meliola* is a genus of specialized parasites that grow on the surface of living plant leaves and fruits, potentially impairing host growth, reducing fruit quality and crop yield, and ultimately causing economic losses. In this study, two new species of *Meliola* from Guizhou Province, China, were identified based on morphological characteristics and multi-gene phylogenetic analyses (nrITS, nrLSU, and nrSSU) using maximum likelihood (ML) and Bayesian inference (BI) to clarify their taxonomic placement. The new species, *Meliola liboense* and *M. lindericola*, were both found colonizing leaves of *Lindera*. Detailed descriptions, illustrations, and a phylogenetic tree showing the phylogenetic positions of the two new species are provided. These findings enrich the taxonomic diversity of *Meliola* and contribute valuable morphological and molecular data to improve species delimitation and classification within Meliolaceae.

## ﻿Introduction

*Meliola* is the largest and the type genus of the family Meliolaceae (Meliolales, Sordariomycetes), comprising 1,765 species (Khan et al. 2025) and accounting for more than 70% of species within the family (Zeng et al. 2022). Species of *Meliola* are obligately biotrophic phytopathogens that form black colonies on living plant surfaces. They establish parasitic relationships with their host plants by forming appressoria on the surface of host epidermal cells, which facilitate adhesion and penetration. From these appressoria, haustoria subsequently develop and extend into the host epidermis or mesophyll cells to absorb nutrients, resulting in a reduction of chlorophyll, starches, sugars, proteins, and amino acids, but without causing pathogenic damage (Hansford 1961; Old et al. 2003; Rodríguez Justavino and Piepenbring 2007; Hongsanan et al. 2015). While exhibiting high host specificity (infecting nearly 200 plant families, particularly Fabaceae), their exact host range requires molecular validation due to potential taxonomic ambiguities (Hansford 1961; Hongsanan et al. 2015). Members of *Meliola* are characterized by branched, dark brown, superficial mycelium with phialides and 2-celled appressoria; dark brown, flattened to subglobose ascomata with 2–4-spored asci; and cylindrical, dark brown ascospores with 3–4 septa. They differ from other genera in Meliolaceae by having hyphal setae on the mycelium (Goos 1974) and by lacking vermiform appendages and setae on the surface of perithecia (Hansford 1961). The genus *Meliola* was first established by Fries in 1825. The description was later revised by Bornet in 1851, with *Meliola psidii* Fr. (Fries 1830) designated as the type species. Gaillard (1892) and Beeli (1920) provided comprehensive studies on the genus, notably introducing digital formulae to facilitate comparisons among genera and species. Subsequently, the taxonomy of *Meliola* was gradually refined through studies focusing on morphological characteristics and systematic placement (Stevens 1927, 1928; Hansford 1961, 1963; Hosagoudar 2008, 2013). As taxonomic understanding progressed, species lacking hyphal setae were reassigned to newly established genera, and these taxonomic revisions significantly contributed to delineating the genus *Meliola* and its morphological characteristics (Stevens 1927, 1928; Hansford 1961). While early classifications placed *Meliola* within Perisporiales or Ascoloculares (Stevens 1927; Nannfeldt 1932), it is now recognized under the order Meliolales, class Sordariomycetes (Lumbsch and Huhndorf 2010).

Because of their biotrophic lifestyles, species of *Meliola* have yet to be grown in culture. Although ascospore germination was successfully induced by Thite (1975) and Goos (1978), subsequent hyphal growth was extremely slow and ultimately ceased, preventing the establishment of in vitro cultures. Molecular data for species of *Meliola* have therefore been obtained by directly extracting DNA from ascomata. However, this method is often complicated by the presence of hyperparasites (Bermúdez-Cova et al. 2022, 2023) and contaminating microorganisms on the colony surface, which can compromise sequencing quality by introducing non-target DNA and reducing the purity of the extracted genetic material. Owing to the difficulties in isolation and molecular sequencing, available molecular data for this genus remain limited. Although the genus *Meliola* currently comprises 2,365 species and 701 varieties, molecular data are available for only 23 species with reliable taxonomic support in GenBank, including the two newly described in this study (accessed 16 April 2025). The taxonomic framework proposed by Hansford (1961), which classifies species primarily based on host specificity and morphological characteristics, is still widely adopted in recent studies (Hongsanan et al. 2015; Zeng et al. 2017, 2022). Given the current paucity of molecular data, species delineation remains largely contingent upon morphological analysis, particularly comparative studies with previously described congeners associated with identical or phylogenetically proximate host plants. Diagnostic morphological traits include the size and shape of perithecia, haustoria, spores, and setae; the arrangement of attachment cells and phialides; and variation in haustorial structure (Hongsanan et al. 2015; Zeng et al. 2022). While a few lineages have received preliminary support from molecular analyses, the overall phylogenetic framework of the genus remains largely morphology-based, lacking a comprehensive and robust molecular systematics foundation.

In this study, we introduce two new species (*M. liboense* and *M. lindericola*) of Meliolaceae, collected from living plant leaves in Guizhou, China. Morphological observations and phylogenetic analyses were conducted to clarify the classification of these species and their phylogenetic relationships with closely related taxa. Detailed descriptions of the morphological features of these species, along with their molecular characterization, are provided.

## ﻿Materials and methods

### ﻿Sample collection and morphological observation

Samples of leaves infected with black mildews were collected from Libo County, Guizhou Province, China, and brought to the laboratory in paper envelopes, then stored in a dry environment. Specimens were examined using a stereomicroscope (Keyence VHX–7000 digital microscope, Japan) to observe and photograph macroscopic features, including colony structure, color, and distribution of ascomata. Microscopic observations and photomicrographs were made from materials mounted in 60% lactic acid using a compound light microscope (Zeiss Axioscope 5, Germany) equipped with an AxioCam 208 color camera and interference contrast optics. Images were captured to document the morphology, color, size, and other characteristics of microstructures such as hyphae, appressoria, setae, ascomata, spores, and phialides. Measurements were taken using the ZEN2 (blue edition) software, and all images used in figures were processed with Adobe Photoshop (version 2022). Study specimens were deposited in the Herbarium of IFRD (International Fungal Research & Development Centre; Institute of Highland Forest Science, Chinese Academy of Forestry, Kunming, China). Index Fungorum numbers were obtained by submitting the species information via the official online registration platform (Index Fungorum 2025).

### ﻿Host plant identification

A preliminary morphological identification of the host plant was performed, followed by molecular biological identification. Morphological identifications were confirmed through consultation with taxonomic experts. Total genomic plant DNA was extracted using a Plant gDNA maxi kit (Biomiga, San Diego, California, USA) in accordance with the manufacturer’s instructions. The partial *rbcL* gene was amplified using the primer pair SI_For and SI_Rev, which were developed by Kress et al. (2009). The 5.8S rDNA along with the internal transcribed spacer (ITS) was amplified with the primer pair ITS1 and ITS4 (White et al. 1990).

### ﻿DNA extraction, PCR amplification, and sequencing

Total genomic fungal DNA was directly extracted from ascomata following the protocol described by Zeng et al. (2018). The 5.8S ribosomal RNA along with the internal transcribed spacer (ITS) was amplified with the primer pair ITS1 and ITS4 (White et al. 1990). The partial large subunit ribosomal RNA (LSU) was amplified with the primer pair LR0R and LR5 (Vilgalys and Hester 1990; Rehner and Samuels 1994). The partial small subunit ribosomal RNA (SSU) was amplified with the primer pair NS1 and NS4 (White et al. 1990). Polymerase chain reactions (PCRs) were performed in a 20 µL reaction mixture containing 17 µL of GoldenStar T6 Super PCR Mix (1.1×), 1 µL of DNA template, and 1 µL each of forward and reverse primers (10 µM/µL). Amplifications were programmed for an initial denaturation step at 95°C, followed by 35 cycles of 30 s at 95°C, 30 s at 54°C (ITS), 50 s at 50°C (LSU), 51°C (SSU), and 50°C (*rbcL*), and 90 s at 72°C, with a final elongation step of 10 min at 72°C. PCR amplification products were assayed via electrophoresis in 1% agarose. The PCR products were then sent to Tsingke Biotechnology Co., Ltd., Beijing, China. The list of primers and PCR conditions for each primer pair is provided in Table 1.

**Table 1.** The PCR conditions and the primers used in this study.

Locus	Primers	Sequence (5’–3’)	PCR cycles	References
ITS	ITS1	TCCGTAGGTGAACCTGCGG	(95°C: 30s, 54 °C:30s, 72°C:60s) × 35 cycles	White et al. (1990)
	ITS4	TCCTCCGCTTATTGATATGC		
LSU	LR0R	ACCCGCTGAACTTAAGC	(94°C: 30s, 51°C: 50s, 72°C:60s) × 35 cycles	Rehner and Samuels (1994)
	LR5	TCCTGAGGGAAACTTCG		Vilgalys and Hester (1990)
SSU	NS1	GTAGTCATATGCTTGTCTC	(95°C: 30s, 51 °C: 50s, 72°C: 60s) × 35 cycles	White et al. (1990)
	NS4	CTTCCGTCAATTCCTTTAAG		
RBCL–a	RBCLf	ATGTCACCACAAACAGAGACTAAAGC	(95°C: 30s, 50 °C: 50s, 72°C: 60s) × 35 cycles	Kress et al. (2009)
	RBCLr	GTAAAATCAAGTCCACCRCG		

### ﻿Phylogenetic analyses

New DNA sequences of each gene, generated from forward and reverse primers, were assembled using BioEdit v.7.2.5 (Hall 1999) to obtain consensus sequences. Phylogenetic analyses were conducted based on datasets including both reference DNA sequences and newly generated DNA sequences using OFPT (Zeng et al. 2023), following the protocol below. Datasets of each gene region were first independently aligned with the “FFT-NS-i” strategy (based on data size) by MAFFT (Katoh and Standley 2013) and trimmed manually. The best-fit nucleotide substitution models for each dataset were then selected according to the Bayesian information criterion (BIC) from 22 common DNA substitution models with rate heterogeneity, using ModelFinder (Kalyaanamoorthy et al. 2017). All datasets were subsequently concatenated with partition information for the following phylogenetic analyses. Maximum likelihood (ML) analysis with 1,000 replicates was performed using ultrafast bootstrap approximation (Hoang et al. 2017) with the SH-like approximate likelihood ratio test (SH-aLRT) (Guindon et al. 2010) implemented in IQ-TREE (Nguyen et al. 2015) The consensus tree was summarized based on the extended majority-rule criterion. Bayesian inference (BI) was performed with two parallel Metropolis-coupled runs (one “cold” chain and three heated chains) using MrBayes (Ronquist et al. 2012).

## ﻿Results

### ﻿Molecular phylogeny

**Figure 1.**
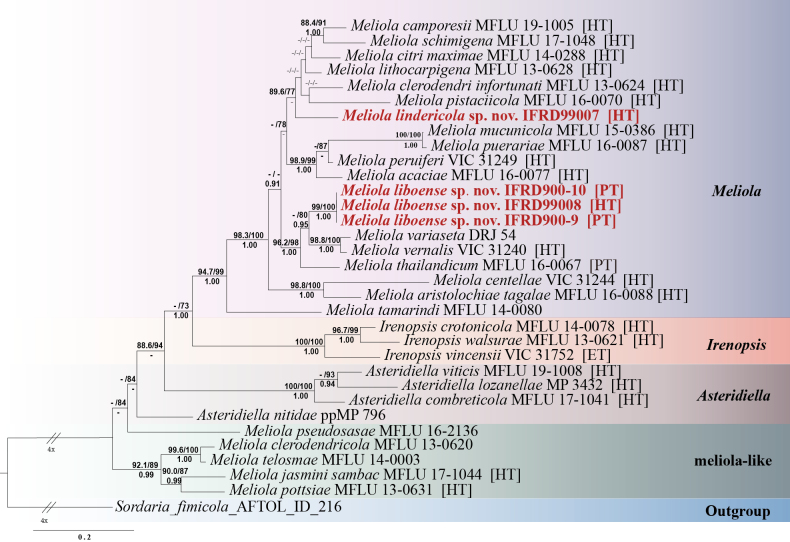
Best-scoring ML tree based on combined LSU, SSU, and ITS sequence data of Meliolaceae, rooted by *Sordaria fimicola*. The SH-aLRT values greater than 80%, BS values greater than 70%, and PP values greater than 0.9 are shown above the branches. Sequences obtained from holotype, paratype, and epitype specimens are marked with HT, PT, and ET, respectively. New sequences obtained in this study are indicated in bold red.

The newly generated sequences were deposited in GenBank, and their accession numbers are provided in Table 2. Phylogenetic analyses for Meliolaceae were performed using ITS, LSU, and SSU loci. The concatenated dataset consisted of 33 strains, with *Sordaria fimicola* designated as the outgroup. The final alignment comprised 2,026 concatenated characters, spanning positions 1–179 (ITS), 180–1,046 (LSU), and 1,047–2,026 (SSU). The combined dataset yielded a best-scoring tree with a final ML optimization likelihood value of –9724.306753. The phylogenetic information used for ML and BI analyses, including model selection and sequence characteristics, is detailed in Table 3. The topology of the tree from BI was similar to that obtained from ML. Given the similarity between the ML and BI topologies, only the ML tree is shown (Fig. 1).

**Table 2.** GenBank accession numbers of DNA sequences used in this study.

Species	Specimen voucher	GenBank accession number			References
		SSU	ITS	LSU	
*Asteridiella combreticola*	MFLU 17-1041	NG_077427	NR_174812	MN747481	Zeng et al. (2022)
*Asteridiella viticis*	MFLU 19-1008	–	NR_174824	NG_079538	Marasinghe et al. (2020)
*Asteridiella lozanellae*	MP 3432	DQ508301	–	DQ508302	Rodríguez Justavino and Piepenbring (2007)
*Asteridiella nitidae*	ppMP 796	–	–	EF094839	Rodríguez Justavino et al. (2015)
*Asteridiella obesa*	VIC 31239	NG_065009	NR_120256	NG_057014	Pinho et al. (2012a)
*Irenopsis crotonicola*	MFLU 14-0078	NG_065668	KY554798	KY554798	Zeng et al. (2017)
*Irenopsis vincensii*	VIC 31752	KC618652	NR_154061	JX096807	Pinho et al. (2012a)
*Irenopsis walsurae*	MFLU 13-0621	NG_228705	NR_154075	KT021648	Zeng et al. (2022)
*Meliola acacia*	MFLU 16-0077	NG_077426	NR_174811	MN747479	Zeng et al. (2022)
*Meliola aristolochiae-tagalae*	MFLU 16-0088	MN747496	–	NG_079535	Zeng et al. (2022)
*Meliola camporesii*	MFLU 19-1005	–	NR_186987	NG_228823	Bhunjun et al. (2022)
*Meliola centellae*	VIC 31244	–	NR_137799	JQ734545	Pinho et al. (2012a)
*Meliola citri-maximae*	MFLU 14-0288	NG_070325	NR_185523	KX458474	Hyde et al. (2016)
*Meliola clerodendricola*	MFLU 13–0620	MN747486	KT021647	KT021647	Zeng et al. (2022)
*Meliola clerodendri-infortunati*	MFLU 13-0624	NG_070324	NR_160479	MF175626	Zeng et al. (2022)
*Meliola jasmini-sambac*	MFLU 17-1044	NG_077428	NR_174813	MN747482	Zeng et al. (2022)
*Meliola lithocarpigena*	MFLU 13-0628	NG_077423	NR_174809	MN747474	Zeng et al. (2022)
*Meliola mucunicola*	MFLU 15-0386	–	KT157534	KT157533	Hongsanan et al. (2015)
*Meliola peruiferi*	VIC 31249	–	–	NG_060294	Pinho et al. (2012b)
*Meliola pistaciicola*	MFLU 16-0070	NG_077425	MN747478	MN747478	Zeng et al. (2022)
*Meliola pottsiae*	MFLU 13-0631	NG_077424	NR_174810	MN747475	Zeng et al. (2022)
*Meliola pseudosasae*	MFLU 16-2136	–	–	KX845434	Hyde et al. (2016)
*Meliola puerariae*	MFLU 16-0087	–	–	NG_079534	Zeng et al. (2022)
*Meliola schimigena*	MFLU 17-1048	–	MN747484	MN747484	Zeng et al. (2022)
*Meliola tamarindi*	MFLU 14-0080	KY554797	KY554799	KY554799	Zeng et al. (2018)
*Meliola telosmae*	MFLU 14-0003	MK103390	MK103389	MK103389	Chethana et al. (2021)
*Meliola thailandicum*	MFLU 16-0067	MN747492	MN747476	MN788606	Hongsanan et al. (2015)
*Meliola variaseta*	DRJ 54	–	–	EF094840	Pinho et al. (2012a)
*Meliola vernalis*	VIC 31240	–	–	JX096808	Pinho et al. (2012a)
*Meliola libosense*	IFRD99008	PQ483902	PQ481147	PQ483770	This Study
*Meliola libosense*	IFRD900-9	PQ483899	PQ481145	PQ483767	This Study
*Meliola libosense*	IFRD900-10	PQ483901	PQ481146	PQ483769	This Study
*Meliola lindericola*	IFRD99007	PQ483900	PQ481148	PQ483768	This Study
*Sordaria fimicola*	AFTOL-ID 216	AY545724	DQ518178	AY545728	Muhammad et al. (2017)

**Table 3.** Summary of phylogenetic parameters used in ML and BI analyses, including the best-fit substitution models, number of nucleotide positions, conserved and variable sites, and other relevant characteristics for each gene region.

Information	Gene		
	ITS	LSU	SSU
Best fit substitution mode	F81+F+R5	GTR+F+I+G4	TIM2e+R2
Number of sequences	25	33	22
Number of characters	179	867	980
Number of constant sites	121 (representing 67.60% of all sites)	525 (representing 60.55% of all sites)	869 (representing 88.67% of all sites)
Number of parsimony informative sites	42	248	50
Number of distinct site patterns	63	387	100
Estimated base frequencies	A = 0.2912	A = 0.2484	equal frequencies
	C = 0.2416	C = 0.216	
	G = 0.2314	G = 0.3152	
	T = 0.2358	T = 0.2203	
Estimated substitution rates	A-C = 1.0000	A-C = 0.8300	A-C = 3.0548
	A-G = 1.0000	A-G = 5.4341	A-G = 6.8999
	A-T = 1.0000	A-T = 1.9822	A-T = 3.0548
	C-G = 1.0000	C-G = 0.3599	C-G = 1.0000
	C-T = 1.0000	C-T = 10.1670	C-T = 15.5297
	G-T = 1.0000	G-T = 1.0000	G-T = 1.0000

Based on the ML and BI analyses, Meliolaceae consists of four generic lineages, represented by the genera *Meliola*, meliola-like, *Asteridiella*, and *Irenopsis*. The genera *Meliola* (20 representative sequences), *Irenopsis* (3), meliola-like (5), and *Asteridiella* (4) form a monophyletic group, with *Meliola* sister to *Irenopsis* and meliola-like sister to *Asteridiella*.

## ﻿Taxonomy

### ﻿ Meliola liboense

Taxon classificationFungiMeliolalesMeliolaceae

Y.Z. Feng & X.Y. Zeng sp. nov.

D96926B7-0C85-5EE5-AF8F-D83A0EFC16B7

Index Fungorum: IF903772

Fig. 2

#### Etymology.

in reference to the location (Libo County) where the specimen was collected.

#### Description.

***Parasitic*** on the surface of living leaves. Colonies 2–7 mm in diameter, hypophyllous, sometimes epiphyllous, black, dense to subdense. ***Hyphae*** superficial, straight to substraight, branched, septate, darker at septa, each cell 21–29 µm (x̄ = 25 µm, n = 10) long, with dark brown setae. ***Hyphal setae*** 132–227 µm long, scattered, denser around ascomata, straight, 2‐dentate, acute. Appressoria 13.2–16.7 × 8.1–12 µm (x̄ = 15.2 × 9.7 µm, n = 20), 2-celled, brown, clavate, straight to curved, formed near septa, alternate to unilateral, antrorse. **Sexual morph: *Ascomata*** up to 120.8 µm in diameter, subdense, scattered, dark brown, globose to subglobose, outer wall rough, with a central ostiole and basal setae. ***Asci*** unitunicate, 2–3-spored, ovoid at young state, asci wall attenuated or broken when mature, without a fixed shape. ***Ascospores*** 39.4–47.6 × 12.6–17.9 µm (x̄ = 42.7 × 16.1 µm, n = 22), hyaline when young, becoming dark brown when mature, 4‐septate, constricted at the septa, rounded at both ends, smooth‐walled. Asexual morph not observed.

**Figure 2.**
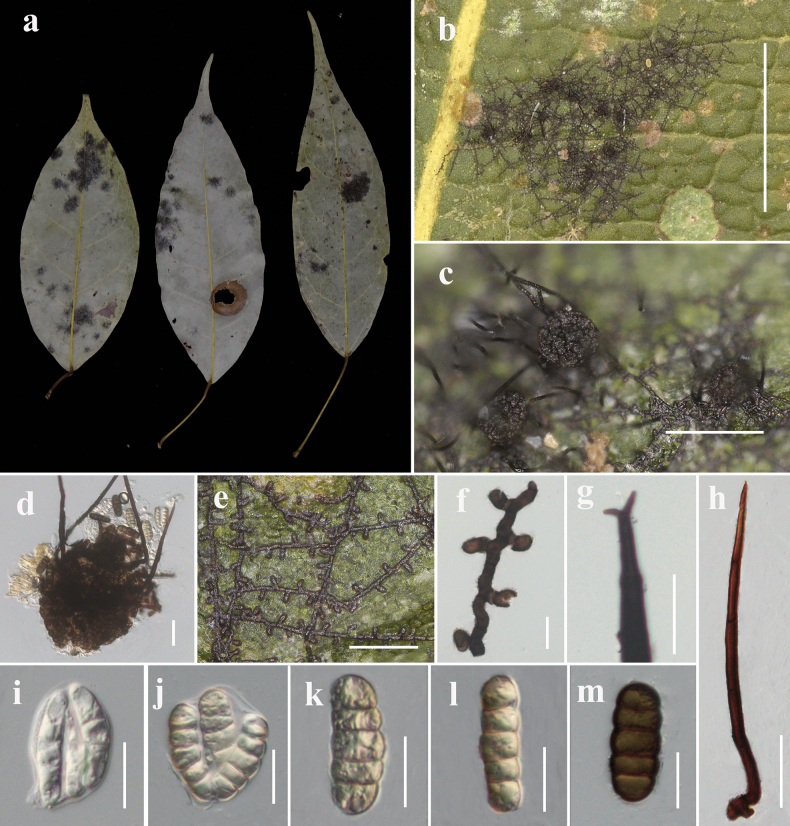
*Meliola liboense* (IFRD99008). a. Fresh leaf of *Lindera* sp.; b. Colonies on the leaf surface; c. Ascomata on the leaf surface; d. Ascomata; e, f. Reticulate hyphae with appressoria and phialides; g. Apex of setae; h. Hyphal setae; i, j. Asci; k-m. Ascospores. Scale bars: 2000 μm (b); 200 μm (c); 50 μm (d); 100 μm (e, i-m); 20 μm (f, g); 50 μm (h).

#### Material examined.

China • Guizhou Province, Libo County, 25°18'32"N, 107°56'10"E; on living leaves of *Lindera* sp.; 13 Jul. 2022; Yu–Wei Liu leg.; IFRD99008 (holotype) • ibid.; IFRD900-10 • ibid.; 8 Apr. 2022; IFRD900-9.

#### Notes.

This collection is typical of *Meliola* in having hyphal setae and represents the first *Meliola* species with DNA sequence data from *Lindera*. There are 6% (541/576 nucleotides, 2 gaps) differences in the LSU region between *M. liboense* and *M. variaseta* and 5% (699/733 nucleotides, 6 gaps) differences in the LSU region between *M. liboense* and *M. vernalis*. However, the *M. vernalis* has larger ascomata (up to 275 μm) and longer hyphal setae (214.5–310 *vs.* 132–227 µm) than *M. liboense* (Pinho et al. 2012b). Although the original description of *M. linderae*, previously reported from the same host, is unavailable for detailed comparison, the present specimen differs clearly from known taxa based on distinct morphological traits and supported by phylogenetic analysis. Therefore, it is described herein as a new species, *M. liboense*.

### ﻿ Meliola lindericola

Taxon classificationFungiMeliolalesMeliolaceae

Y.Z. Feng & X.Y. Zeng sp. nov.

6EB2A266-62F5-5454-A0AC-518792A46F02

Index Fungorum: IF903770

Fig. 3

#### Etymology.

in reference to the host species name.

#### Holotype.

IFRD99007.

#### Description.

***Parasitic*** on the surface of living leaves. Colonies 10–12 mm in diameter, hypophyllous, sometimes epiphyllous, black, dense to subdense. ***Hyphae*** superficial, straight to substraight, branched, septate, darker at septa, each cell (18-) 21–45 µm (x̄ = 40 µm, n = 20) long, with dark brown setae. ***Hyphal setae*** 121–162 µm long, scattered, denser around ascomata, straight, with obtuse tip. ***Asci*** unitunicate, 2–3-spored, ovoid at young state, asci wall attenuated or broken when mature, without a fixed shape. ***Appressoria*** 22.3–29.8 × 11–16.5 µm (x̄ = 26.1 × 14 µm, n = 20), 2–celled, brown, clavate, straight to curved, formed near septa, alternate to unilateral, antrorse. **Sexual morph: *Ascomata*** up to 120 µm in diameter, subdense, scattered, dark brown, globose to subglobose, outer wall rough, with a central ostiole and basal setae. Asci not observed. ***Ascospores*** 21.3–25.5 × 6.8–9.3 µm (x̄ = 21.7 × 8.2 µm, n = 30), hyaline ascospores 19.8–21.1 × 4.4–8.2 µm (x̄ = 20.6 × 5.7 µm, n = 6), cylindrical or oblong, hyaline when young, becoming dark brown when mature, 2–4-septate, constricted at the septa, rounded at both ends, smooth-walled. **Asexual morph**: Phialides 14.3–19.3 × 8.3–13 µm (x̄ = 16.4 × 9.8 µm, n = 6), opposite to unilateral, few mixed with appressoria, ampulliform.

#### Material examined.

China • Guizhou Province, Libo County; 25°15'10"N, 107°44'6"E; on living leaves of *Lindera* sp.; 12 Jul. 2022; Yu-Wei Liu leg.; IFRD99007 (holotype).

#### Notes.

This collection is typical of *Meliola* in having hyphal setae (Goos 1974). Phylogenetically, this new collection clusters with *M. pistaciicola*, but with 10% (186/203, 87/99 nucleotides, 5 gaps), 8% (773/837 nucleotides, 9 gaps), and 1% (994/999 nucleotides, no gaps) differences in ITS, LSU, and SSU regions, respectively. Moreover, *M. lindericola* is morphologically different in having shorter hyphae setae (162 *vs* 700 µm), smaller ascomata (120 *vs* 230 µm), ascospores (21.3–25.5 × 6.8–9.3 *vs* 43–55 × 17–25 μm), and phialides (14.3–19.3 × 8.3–13.0 *vs* 21–31 × 9–12 μm), and the phialides are opposite (Zeng et al. 2022). Besides, *M. lindericola* has larger appressoria (22.3–29.8 × 11.0–16.5 *vs* 13.2–16.7 × 8.1–12 μm), smaller ascospores (21.3–25.5 × 6.8–9.3 *vs* 39.4–47.6 × 12.6–17.9 μm), shorter setae (121–162 *vs* 132–227 μm), and an obtuse tip than *M. liboense*. Although the original description of *M. linderae* from the same host is unavailable for direct comparison, the distinct morphological features and molecular data obtained from our specimens support its recognition as a new species.

**Figure 3.**
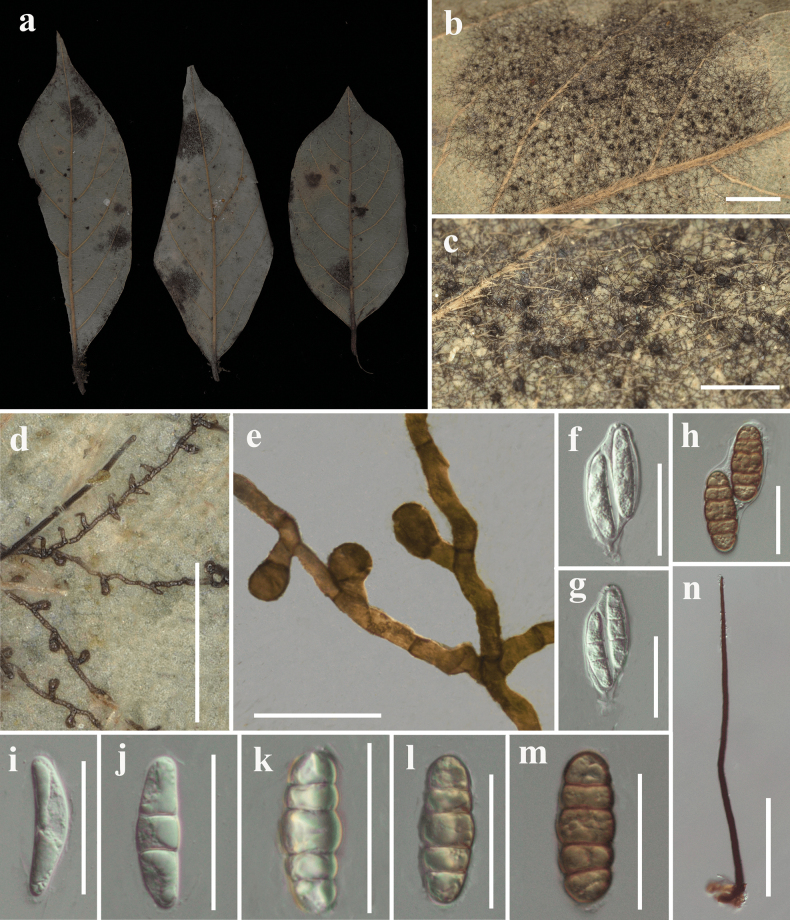
*Meliola lindericola* (IFRD99007). a. Fresh leaf of *Lindera* sp.; b. Colonies on the leaf surface; c. Ascomata on the leaf surface; d, e. Reticulate hyphae with appressoria and phialides; f-h. Asci from young to mature; i-m. Ascospores; n. Hyphal setae. Scale bars: 2000 μm (b); 1000 μm (c); 200 μm (d); 50 μm (e); 20 μm (f-m); 50 μm (n).

## ﻿Discussion

Morphological and phylogenetic analyses of the collected specimens confirm the distinctiveness of *M. liboense* and *M. lindericola* as new species. Species delimitation was supported through an integrative approach combining detailed morphological examination, host information, and phylogenetic analysis. Both new species form independent lineages in the phylogenetic trees and show consistent differences from closely related taxa, confirming their recognition as distinct species. These findings enrich the known species diversity of *Meliola* and provide valuable molecular data for future studies within the family.

Currently, the classification of the genus *Meliola* still follows the system proposed by Hansford (1961), which is primarily based on host specificity and morphological characteristics. Since most species in Meliolaceae are known only from their sexual morphs, species delimitation heavily relies on traits such as the length and apical morphology of setae; the arrangement and dimensions of appressoria and phialides; the size of the ascomata; and the size and number of septa in ascospores. For example, *M. acaciae* and *M. peruiferi* can be distinguished based on differences in colony position, ascomata size, the arrangement of phialides and appressoria, and the apical morphology of setae (Zeng et al. 2022), while *M. hosagoudarii* and *M. litseicola* differ notably in the size and shape of ascospores (Song et al. 2002). Additionally, *M. pistaciicola* and *M. clerodendri-infortunati* are differentiated in that the latter has epiphyllous colonies, shorter hyphal cells, smaller appressoria and ascomata, larger ascospores, and simple, acute setae (Zeng et al. 2022). However, morphological characteristics often exhibit limited interspecific variation, and the considerable species diversity within the family further complicates accurate and efficient identification through traditional taxonomic methods.

In this study, only published sequences were included in the phylogenetic analyses to ensure data reliability. To avoid introducing systematic bias, *Endomeliola dingleyae* was excluded from the ingroup, as its phylogenetic position is markedly distant from other members of Meliolaceae. Zeng et al. (2022) found that *Meliola* has traditionally been defined by the presence of setae, but it has diverged into two distinct phylogenetic lineages through co-evolution with angiosperms. Preliminary analyses suggest that this divergence may be related to the density of setae surrounding the ascomata. In addition, *Asteridiella* was revealed to be polyphyletic; phylogenetic analysis placed *Asteridiella obesa* within the clade of *Meliola* (Zeng et al. 2022). These findings imply that certain morphological characteristics, such as setal position, may not consistently correspond with phylogenetic relationships, indicating that the current morphology-based classification system may require further refinement. Although molecular sequence data for Meliolaceae are available in NCBI, some lack corresponding publications (e.g., KR706162, KC618648). Future taxonomic studies should combine molecular phylogenetic analyses with a critical reassessment of morphological traits to establish a more natural and robust classification for the family.

## Supplementary Material

XML Treatment for Meliola liboense

XML Treatment for Meliola lindericola

## ﻿Additional information

### Use of AI

No use of AI was reported.

### Author ORCIDs

Yuzhe Feng https://orcid.org/0009-0000-8832-7355

Yuwei Liu https://orcid.org/0000-0002-0346-149X

Sinang Hongsanan https://orcid.org/0000-0003-0550-3152

Entaj Tarafder https://orcid.org/0000-0002-3680-3433

Ting-Chi Wen https://orcid.org/0000-0003-1744-5869

Xiang-Yu Zeng https://orcid.org/0000-0003-1341-1004

Fengquan Liu https://orcid.org/0000-0001-9325-1500

### Data availability

All of the data that support the findings of this study are available in the main text.
